# Supplementary material for: Yeast Models of Amyotrophic Lateral Sclerosis Type 8 Mimic Phenotypes Seen in Mammalian Cells Expressing Mutant VAPBP56S
Source: Biomolecules. 2023 Jul 19;13(7):1147. doi: 10.3390/biom13071147 (PMC10377116; doi:10.3390/biom13071147)
Supplement: Supplementary file 1 [file biomolecules-13-01147-s001.zip › biomolecules-2424134-supplementary-conversion/Supplementary Files/Supplementary File 1.pdf]

Supplementary File 1: Coding sequences of yeast *SCS2* and human *VAPB* used in this study

1-1: *SCS2*^{WT}
1-2: *scs2*$^{P51S,\ P58S}$
1-3: *VAPB*^{WT}
1-4: *VAPB*^{P56S}

For mutant alleles, nucleotide substitutions are noted in lowercase. Underlined sequences correspond to missense mutations resulting in *scs2*$^{P51S,\ P58S}$ and *VAPB*^{P56S} alleles. Red text in the *scs2*$^{P51S,\ P58S}$ coding sequence indicate PvuI and NheI sites that were intentionally introduced with silent mutations to facilitate detection of the mutant allele in future applications.

1-1: *SCS2(WT)*

ATGTCTGCTGTTGAAATTTCCCCTGACGTGTTGGTGTATAAGTCTCCATTAACGGAGCAATCGACAGAATACGCTAG
TATTTCCAACAATTCAGACCAAACCATTGCATTTAAGGTCAAGACCACAGCCCCAAAGTTTTACTGCGTGAGACCAA
ACGCCGCAGTTGTTGCTCCAGGTGAAACAATCCAGGTTCAAGTTATCTTCCTGGGTTTGACTGAAGAGCCTGCCGCA
GACTTTAAGTGCCGTGACAAGTTCCTGGTTATCACGTTGCCTTCTCCTTACGACCTCAATGGCAAGGCTGTTGCAGA
TGTTTGGTCCGACTTGGAGGCTGAGTTCAAGCAACAGGCTATCTCTAAAAAGATAAAAGTCAAATATTTGATAAGTC
CAGATGTACACCCAGCACAGAACCAAAATATACAGGAAAACAAGGAAACTGTTGAACCAGTAGTCCAAGACAGTGAG
CCAAAGGAAGTGCCTGCTGTGGTAAACGAAAAGGAGGTCCCTGCTGAACCGGAAACTCAACCTCCCGTCCAAGTAAA
GAAAGAAGAGGTCCCACCTGTCGTTCAGAAAACCGTTCCTCATGAAAATGAGAAGCAAACGTCCAACAGCACTCCGG
CCCCACAGAACCAGATAAAGGAAGCCGCAACCGTACCTGCAGAAAATGAATCATCCAGCATGGGTATATTCATATTG
GTTGCACTCCTTATCTTGGTTTTAGGATGGTTCTACAGATAA

1-2: *scs2(P51S, P58S)*

ATGTCTGCTGTTGAAATTTCCCCTGACGTGTTGGTGTATAAGTCTCCATTAACGGAGCAATCGACAGAATACGCTAG
TATTTCCAACAATTCAGACCAAACCATTGCATTTAAGGTCAAGACCACAGCCCCAAAGTTTTACTGCGTGcGAtCgA
ACGCCGCAGTTGTTGCTagcGGTGAAACAATCCAGGTTCAAGTTATCTTCCTGGGTTTGACTGAAGAGCCTGCCGCA
GACTTTAAGTGCCGTGACAAGTTCCTGGTTATCACGTTGCCTTCTCCTTACGACCTCAATGGCAAGGCTGTTGCAGA
TGTTTGGTCCGACTTGGAGGCTGAGTTCAAGCAACAGGCTATCTCTAAAAAGATAAAAGTCAAATATTTGATAAGTC
CAGATGTACACCCAGCACAGAACCAAAATATACAGGAAAACAAGGAAACTGTTGAACCAGTAGTCCAAGACAGTGAG
CCAAAGGAAGTGCCTGCTGTGGTAAACGAAAAGGAGGTCCCTGCTGAACCGGAAACTCAACCTCCCGTCCAAGTAAA
GAAAGAAGAGGTCCCACCTGTCGTTCAGAAAACCGTTCCTCATGAAAATGAGAAGCAAACGTCCAACAGCACTCCGG
CCCCACAGAACCAGATAAAGGAAGCCGCAACCGTACCTGCAGAAAATGAATCATCCAGCATGGGTATATTCATATTG
GTTGCACTCCTTATCTTGGTTTTAGGATGGTTCTACAGATAA

1-3: *VAPB(WT)*

ATGGCGAAGGTGGAGCAGGTCCTGAGCCTCGAGCCGCAGCACGAGCTCAAATTCCGAGGTCCCTTCACCGATGTTGT
CACCACCAACCTAAAGCTTGGCAACCCGACAGACCGAAATGTGTGTTTTAAGGTGAAGACTACAGCACCACGTAGGT
ACTGTGTGAGGCCCAACAGCGGAATCATCGATGCAGGGGCCTCAATTAATGTATCTGTGATGTTACAGCCTTTCGAT
TATGATCCCAATGAGAAAAGTAAACACAAGTTTATGGTTCAGTCTATGTTTGCTCCAACTGACACTTCAGATATGGA
AGCAGTATGGAAGGAGGCAAAACCGGAAGACCTTATGGATTCAAAACTTAGATGTGTGTTTGAATTGCCAGCAGAGA
ATGATAAACCACATGATGTAGAAATAAATAAAATTATATCCACAACTGCATCAAAGACAGAAACACCAATAGTGTCT
AAGTCTCTGAGTTCTTCTTTGGATGACACCGAAGTTAAGAAGGTTATGGAAGAATGTAAGAGGCTGCAAGGTGAAGT
TCAGAGGCTACGGGAGGAGAACAAGCAGTTCAAGGAAGAAGATGGACTGCGGATGAGGAAGACAGTGCAGAGCAACA
GCCCCATTTCAGCATTAGCCCCAACTGGGAAGGAAGAAGGCCTTAGCACCCGGCTCTTGGCTCTGGTGGTTTTGTTC
TTTATCGTTGGTGTAATTATTGGGAAGATTGCCTTGTAG

1-4: *VAPB(P56S)*

ATGGCGAAGGTGGAGCAGGTCCTGAGCCTCGAGCCGCAGCACGAGCTCAAATTCCGAGGTCCCTTCACCGATGTTGT
CACCACCAACCTAAAGCTTGGCAACCCGACAGACCGAAATGTGTGTTTTAAGGTGAAGACTACAGCACCACGTAGGT
ACTGTGTGAGGtCCAACAGCGGAATCATCGATGCAGGGGCCTCAATTAATGTATCTGTGATGTTACAGCCTTTCGAT
TATGATCCCAATGAGAAAAGTAAACACAAGTTTATGGTTCAGTCTATGTTTGCTCCAACTGACACTTCAGATATGGA
AGCAGTATGGAAGGAGGCAAAACCGGAAGACCTTATGGATTCAAAACTTAGATGTGTGTTTGAATTGCCAGCAGAGA
ATGATAAACCACATGATGTAGAAATAAATAAAATTATATCCACAACTGCATCAAAGACAGAAACACCAATAGTGTCT
AAGTCTCTGAGTTCTTCTTTGGATGACACCGAAGTTAAGAAGGTTATGGAAGAATGTAAGAGGCTGCAAGGTGAAGT
TCAGAGGCTACGGGAGGAGAACAAGCAGTTCAAGGAAGAAGATGGACTGCGGATGAGGAAGACAGTGCAGAGCAACA
GCCCCATTTCAGCATTAGCCCCAACTGGGAAGGAAGAAGGCCTTAGCACCCGGCTCTTGGCTCTGGTGGTTTTGTTC
TTTATCGTTGGTGTAATTATTGGGAAGATTGCCTTGTAG
