# Supplementary material for: Yeast Models of Amyotrophic Lateral Sclerosis Type 8 Mimic Phenotypes Seen in Mammalian Cells Expressing Mutant VAPBP56S
Source: Biomolecules. 2023 Jul 19;13(7):1147. doi: 10.3390/biom13071147 (PMC10377116; doi:10.3390/biom13071147)
Supplement: Supplementary file 1 [file biomolecules-13-01147-s001.zip › biomolecules-2424134-supplementary-conversion/Supplementary Files/Supplementary File 2.pdf]

Supplementary File 2: Pairwise alignments of human ALS-linked proteins with yeast homologs

Yellow highlight: identical amino acid
Blue highlight: similar amino acid
Red text: dissimilar amino acid, ALS-linked in human

2-1: ALS1 (*SOD1*)
2-2: ALS4 (*SETX*)
2-3: ALS8 (*VAPB*)
2-4: ALS11 (*FIG4*)
2-5: ALS13 (*ATXN2*)
2-6: ALS14/FTD-ALS6 (*VCP*)
2-7: ALS15 (*UBQLN2*)
2-8: ALS16 (*SIGMAR1*)
2-9: ALS17/FTD-ALS7 (*CHMP2B*)
2-10: ALS18 (*PFN1*)
2-11: ALS20 (*HNRNPA1*)
2-12: ALS22 (*TUBA4A*)
2-13: ALS25 (*KIF5A*)
2-14: ALS26 (*TIA1*)
2-15: ALS27 (*SPTLC1*)
2-16: FTD-ALS2 (*CHCHD10*)
2-17: FTD-ALS5 (*CCNF*)

2-1: CLUSTAL O(1.2.4) multiple sequence alignment **[ALS1]**

```
hsSOD1      MATKAVCVLKGDGPVQGIINFEQKESNGPVKVWGSIKG-LTEGLHGFHVHEFGDNTAGCT     59
scSOD1      -MVQAVAVLKGDAGVSGVVKFEQASESEPTTVSYEIAGNSPNAERGFHIHEFGDATNGCV     59
              .:**.*****. *.*:::*** ... *..*  .* *   :. :***:***** * **.

hsSOD1      SAGPHFNPLSRKHGGPKDEERHVGDLGNVTADKDGVADVSIEDSVISLSGDHCIIGRTLV     119
scSOD1      SAGPHFNPFKKTHGAPTDEVRHVGDMGNVKTDENGVAKGSFKDSLIKLIGPTSVVGRSVV     119
            ********:.:.**.*.** *****:***.:*::***. *::**:*.* *  .::**::*

hsSOD1      VHEKADDLGKGGNEESTKTGNAGSRLACGVIGIAQ    154
scSOD1      IHAGQDDLGKGDTEESLKTGNAGPRPACGVIGLTN    154
            :*   ******..*** ****** * ******:::
```

2-2: CLUSTAL O(1.2.4) multiple sequence alignment **[ALS4]**

```
hsSETX      MSTCCWCTPGGASTIDFLKRYASNTPSGE----FQTA--DEDLC--YCLECVAEYHKARD     52
scSEN1      --------------------MNSNNPDNNNSNNINNNNKDKDIAPNSDVQLATVYTKAKS     40
                                  **.*..:    ::.   *:*:.    :: .: * **:.

hsSETX      ELPFLHEVLWELETLRLINHFEKSMKAEIGDDDELYIVDNNGEMPLFDITGQDFENKLRV     112
scSEN1      YIPQIEQVYQG-------------------------------------------------     51
             :* :.:*

hsSETX      PLLEILKYPYLLLHERVNELCVEALCRMEQANCSFQVFDKHPGIYLFLVHPNEMVRRWAI     172
scSEN1      ---------------------------------------TNPN----------------I     56
                                                   .:*.                *

hsSETX      LTARNLGKVDRDDYYDLQEVLLCLFKVIELGLLESPDIYTSSVLEKGKLILLPSHMYDTT     232
scSEN1      QEAKLLG-----------ELLQVL-A----EVPKGTHLFCDPILEPISIFSLTIFSFNE-     99
              *: **           *:*  *       : :. .:: . :**  .:: *  . ::

hsSETX      NYKSYWLGICML--LTILEEQAMDSLLLGSDKQNDFMQSILHTMEREADDD------SVD     284
scSEN1      EATATWLKNHFNPILSVCD-KCILNFARGKCK---MLQH--FAIQRHVPHEHVAKFNDIV     153
            : .: **   :   *:: : :.: .:  *. *   ::*   .:::*.. .:      .:

hsSETX      PFWPALHCFMVIL-----DRLG--------SKVWGQLMDPIVA---------FQTIINNA     322
scSEN1      CQWRVEAVFPILRNISVNDNTGINITNEIETAMYECLCNPHMLRLNKQLKATFEAIF-KF     212
              * .   * ::      *. *        : ::  * :* :          *::*: :

hsSETX      SYNREIRHIRNSSVRTKLEPESYLDDMVTCSQIVYNYNPEKTKKDSGWRTAICPDYCPNM     382
scSEN1      FYDTKHR---LLDVTNPLSIKTFISGVIFC------W-CEGSKEENEWSRAFLKDLYSRN     262
             *: : *     .* . *. ::::..:: *      :  * :*::. *  *:  *   .

hsSETX      YEEMETLASVLQSDIGQDMRVHNSTFLWFIPFVQSLMDLKDLGVAYIAQVVNHLYSEVKE     442
scSEN1      FHI----------------------------------NL---------------------     267
            :.                                   :*

hsSETX      VLNQTDAVCDKVTEFFLLILVSVIELHRNKKCLHLLWV-SSQQWVEAVVKC---------     492
scSEN1      ----SNLTPDIIEEVY----------------IHILFLQNPANWTEIVVSQFWSRLLPVF     307
                :: . * : *.:                :*:*:: .  :*.* **.

hsSETX      AKLPTTAFTRSSEKSSG-NCSKGTAMISSLSLHSMPSNSVQLAYVQLIRSLLKEGYQLGQ     551
scSEN1      NLFDKDVFIEYFQVPKNVESLKKTFKFPLEPIFKMWYNHLSKSYHDKPLDFLLRGLTMFL     367
              : . .* .  :  .. :. * *  :    :..*  * :. :* :   .:* .*  :

hsSETX      QSLCKRFWDKLNLFLRGNLSLGWQLTSQETHELQSCLKQIIRNIKFKAPPCNTFVDLTSA     611
scSEN1      NKFGSEFWSKIEPFTFHSI-----------------LDIIFNR-----------------     393
            :.: ..**.*::  *   .:                  *. *:..

hsSETX      CKISPASYNKEESEQMGKTSRKDMHCLEASSPTFSKEPMKVQDSVLIKADNTIEGDNNEQ     671
scSEN1      -------------------------------DSFPIKLIKIQDNPIVEH-------QTE-     414
                                            :*  : :*:**. :::        :.*

hsSETX      NYIKDVKLEDHLLAGSCLKQSSK--NIFTE-RAEDQIKISTRKQ-KSVKEISSY-----T     722
scSEN1      VYFQ--------LTGSVTDLLSWTLPFYHALSPSKRIQMVRKVSMAFLRIIANYPSLKSI     466
             *::        *:**  .  *    ::     ..:*::  : .   ::  *:.*

hsSETX      PKDCTSRNGPERGCDRGIIVSTRLLTDSSTDALEKVSTS---NEDFSLKDDALAKTSKRK     779
scSEN1      PKACLMNSA--------------------TALLRAVLTIKENERAMLYKNDEFE------     500
            ** *  ...                    *  *. * *    :. :  *:* :

hsSETX      TKVQKDEICAKLSHVIKKQHRKSTLVDNTINLDEN-LTVSNIESFYSRKDTGVQ---KGD     835
scSEN1      -------------TVLLTKTDSRALLNNPLIQDIIIRSASNPNDFYPGLGAASASVATST     547
                          *: .:  . :*::* :  *    :.** :.**   .:.     ..
```

```
hsSETX      GFIHNLSLDP------SGVLDDKNGEQKS----QNNVLPKEKQLKNEELVIFSFHENNCK     885
scSEN1      MMVLAECIDFDILLLCHRTFKLYSGKPISEIPISTNVLEN----VTNKIDLRSFHDGPLL     603
             ::   .:*          .:.  .*:  *     ..*** :    .::: : ***:.

hsSETX      IQEFHVDGKELIPFTEMTNASEKKSSPFKDLMTVPESRDEE-M---SNSTSVIYSNLTRE     941
scSEN1      AKQLLVSLK-----------------NINGLLIVPSNTAVAEAHNALNQKFLLLSTRLME     646
             ::: *. *                 ::.*: **..          *.. :: *.   *

hsSETX      QAPDISPKS--DTLTDSQIDRDLHKLSLLAQASVITFPSDSPQNSSQLQRKVKEDKRCFT     999
scSEN1      KFADILPGQLSKILADEDASQGFW------------------------SCIFSSDKHLYQ     682
            :  ** * .  . *:*.: .:.:                          .  ...**: :

hsSETX      ANQN------NVGDTSRGQVIIISDSDDDDDERI-LSLEKLTKQ--DKICLEREHP----     1046
scSEN1      AATNILYNTFDVEGRLEGILAILNSNLTVNLKNINVMLQRLINCEFYEPCPRAVRVLMDV     742
            *  *      :* .  .* : *:...   : :.* : *::* :    : * .  :

hsSETX      -EQHVSTVNSK-EEKNPVKEEKTETLFQFEESDSQCFEFESSSEVF-SVWQDHPDDNNSV     1103
scSEN1      VSAFVDPISGVFANFQTLKSQNTE--KEFLKFWESCWLFLDTIYKFTLKWASKYDYS-EL     799
             . .*. :..   : : :*.::**   :* :  ..*: * .:   *   * .: * . .:

hsSETX      QDGEKKCLAPIANTTNGQGCTDYVSEVVKKGAEGIEEHTRPRSISVEEFCEIEVKKPKRK     1163
scSEN1      E--------------------NFTKDTL--------DLSRSLVDSFREFSDILHDQT---     828
            :                    ::..:.:        : :*    *..**.:*  .:

hsSETX      RSEKPMAEDPVRPSSSVRNEGQSDTNKRELVGN---DFKSIDRRTSTPNSRIQRATTVSQ     1220
scSEN1      ---------------------------KNLLLNVLETFKNMLYWL-----------RL--     848
                                       ::*: *    **.:                :

hsSETX      KKSSKLCTCTEPIRKVPVSKTPKKTHSDAKKRQNRSSNYLSCRTTPAIVPPKKFRQCPEP     1280
scSEN1      SDEVLLESCVRLI-----------------------------------------------     861
            ...  * :*.. *

hsSETX      TSTAEKLGLKKGPRKAYELSQRSLDYVAQLRDHGKTVGVVDTRKKTKLISPQNLSVRNNK     1340
scSEN1      ------------------------------------------------ISTSDLAHEKHV     873
                                                            ** .:*: .::

hsSETX      KLLTSQELQMQRQIRPKSQKNRRRLSDCESTDVKRAGSHTAQNSDVFVPESDRSDYNCTG     1400
scSEN1      KV-DDSLVEMMAKYASKAKRFSNKLTEQQA-------SEILQKAKIFNK-----------     914
            *:  .. ::*  :   *:::  .:*:: ::       *.  *::.:*

hsSETX      GTEVLANSNRKQLIKCMPSEPETIKAKHGSPATDDACPLNQCDSVVLNGTVPTNEVIVST     1460
scSEN1      -----------ALTEEVATEAENYRKEKE------LSRLG--KVIDLTDSVPASPSLSPS     955
                       * : : :* *. : ::        . *.  . : *..:**:.  :  :

hsSETX      SEDPLGGGDPTARHIEMAALKEGEPDSSSDAEEDNLFLTQNDPEDMDLCSQMENDNYKLI     1520
scSEN1      LSSTIA---------------------SSSAESRADYLQRK----------------ALS     978
             .. :.                     **.**.   :* ::                *

hsSETX      ELIHGKDTVEVEEDSVSRPQLESLSGTKCKYKDCLETTKNQGEYCPKHSEVKAADEDVFR     1580
scSEN1      SSITGRP-------RVAQPKITSFGTFQSSANAKLHRTKPV----KPLSKMELARMQLLN     1027
            . * *:        *::*:: *:.  :.. :  *. **         *::: *  :::.

hsSETX      KPGLPPPASKPLRPTTKIFSSKST-SRIAGLSKSLETSSALSPSLKNKSKGIQSILKVPQ     1639
scSEN1      NRVVHPPSAPAFHTKSRGLSNKNDDSSSEESDNDIESARELFAIAKAKGKGIQTVDINGK     1087
            :  : **::  :: .:: :*.*.  *      .:.:*::  *    * *.****::    :

hsSETX      P-----VPLIAQKPVGEMKNSCNVLHPQSPNNSNRQGCKVPFGESKYFPSSSPVNILLSS     1694
scSEN1      VVKRQTAAELAKQELEHMRKR---------------------------------------L     1109
                  .  :*:: : .*::

hsSETX      QSVSDTFVKEVLKWKYEMFLNFGQCGPPASLCQSISRPVPVRFHNYGDYFNVFFPLMVLN     1754
scSEN1      NVDMNPLYEIILQWDYTRNSEYPDDEPIGNY-----SDVKDFFNSPADYQKVMKPLLLLE     1164
            :   : : : :*:*.*    :: :  * ..        *   *:. .** :*: **::*:
```

```
hsSETX      TFETVAQEWLNSPNRENFYQLQV----RKFPADYIKYWE-F-AVYLEECELAKQLYPKEN    1808
scSEN1      SWQGLCS----SRDREDYKPFSIIVGNRTAVSDFYDVYASVAKQVIQDCGISE------S    1214
            ::: :..    * :**::  :.:    *.  :*: . :  .    :::* :::      .

hsSETX      DLVFLAPERINEEKKDTERNDIQDLHEYHSGYVHKFRRTSVMRNGKTECYLSIQTQENFP    1868
scSEN1      DLIVMAYLPDFRP---DKRLSSDDFKKAQHTCLAKVRTLKNTKGGNVDVTLRIHRNHSFS    1271
            **:.:*     .     :* . :*::: :    : *.*  .  :.*:.:  * *: :..*

hsSETX      AN--LNELVNCIVISSLVTTQRKLKAMSLLGSRNQLARAVLNPNPMDFCTKDLLTTTSER    1926
scSEN1      KFLTLRSEIYCVKVMQMTTIEREYSTLEGLEYYD-LVGQILQAKPSPPVNVD-----AAE    1325
                *.. : *: : .:.* :*: .::. *   : *.  :*: :*    . *     : .

hsSETX      IIAYLRDFNEDQKKAIETAYAMVKHSPSVAKICLIHGPPGTGKSKTIVGLLYRLLTENQR    1986
scSEN1      IETVKKSYKLN-----TSQAEAIVNSVSKEGFSLIQGPPGTGKTKTILGIIGYFLSTKNA    1380
            * :  :.:: :       :    : :* *   :.**:*******:***:*::  :*: ::

hsSETX      KG---------HSDENSNAKIKQNRVLVCAPSNAAVDELMKKIILEFKEKCKDKKNPLGN    2037
scSEN1      SSSNVIKVPLEKNSSNTEQLLKKQKILICAPSNAAVDEICLRLKSGVYDKQ-------GH    1433
            ..          :...*::  :*::::*:**********:  ::   . :*        *:

hsSETX      CGDINLVRLGPEKSINSEVLKFSLDSQVNHRMKKE---------LPSHV-QAMHKRKEFL    2087
scSEN1      QFKPQLVRVGRSDVVNVAIKDLTLEELVDKRIGERNYEIRTDPELERKFNNAVTKRRELR    1493
              . :***:*  .. :*   : .::*:. *::*: :.          *  :. :*: **:*:

hsSETX      DYQLDELSRQRALCRGGR-----EIQRQELDENISKVSKERQELASK----IKEVQGRPQ    2138
scSEN1      -GKLDSESGNPESPMSTEDISKLQLKIRELSKIINELGRDRDEMREKNSVNYRNRDLDRR    1552
              :**. * :      . .     ::: :**.: *.::.::*:*: .*     :: :   :

hsSETX      KTQSIIILESHIICCTLSTSGGLLLESAFRGQGGVPFSCVIVDEAGQSCEIETLTPLIHR    2198
scSEN1      NAQAHILAVSDIICSTLSGSAHDVL-----ATMGIKFDTVIIDEACQCTELSSIIPLRYG    1607
            ::*: *:  *.***.*** *.  :*      .  *: *. **:*** *. *:.:: ** :

hsSETX      CNKLILVGDPKQLPPTVISMKAQEYGYDQSMMARFCRLLEENVEHNMISRLPILQLTVQY    2258
scSEN1      GKRCIMVGDPNQLPPTVLSGAASNFKYNQSLFVRMEKN------------SSPYLLDVQY    1655
             :: *:****:******:*  *.:: *:**::.*: :                  * ***

hsSETX      RMHPDICLFPSNYVYNRNLKTNRQTEAIRC---SSDWPFQPYLVFDVGDGSERR--DNDS    2313
scSEN1      RMHPSISKFPSSEFYQGRLKDGPGMDILNKRPWHQLEPLAPYKFFDIISGRQEQNAKTMS    1715
            ****.*. ***. .*: .** .   : :.     .  *: ** .**: .* :.:  .. *

hsSETX      YINVQEIKLVMEIIKLIKDKR-KDV-SFRNIGIITHYKAQKTMIQKDLDKEFDRK--GPA    2369
scSEN1      YTNMEEIRVAIELVDYLFRKFDNKIDFTGKIGIISPYREQMQKMRKEFARYFGGMINKSI    1775
            * *::**::.:*::. :  *  :.:    :****: *: *   ::*:: : *.

hsSETX      EVDTVDAFQGRQKDCVIVTCVRANSIQGSIGFLASLQRLNVTITRAKYSLFILGHLRTLM    2429
scSEN1      DFNTIDGFQGQEKEIILISCVRADDTKSSVGFLKDFRRMNVALTRAKTSIWVLGHQRSLA    1835
            :.:*:*.***::*: ::::****:. :.*:*** .::*:**::**** *:::*** *:*

hsSETX      ENQHWNQLIQDAQKRGAIIKTCDKNYRHDAVKILKLKPVLQRSLTHPPTIAPEGSRPQGG    2489
scSEN1      KSKLWRDLIEDAKDRSCLAYACSGFLDPRNNRAQSI---L-RKFNVPV---PSEQEDDYK    1888
            :.: *.:**:**:.*..:  :*.         :  .:   * *.:. *    *. .. :

hsSETX      LPSSKLDSGFAKTSVAAS--LYHTPSDSKEITLTVTSKDPER------PPVHDQLQDP--    2539
scSEN1      LPMEYITQGPDEVKSNKDTKKRRVVDEGEEADKAVKKKKEKKKEKKKSKADDKKKNNKK    1948
            ** . : .*  :..    .    :. .:.:*   :*..*. *:         ..*: ::

hsSETX      --RLLKRMGIEVKGGIFLWDPQPSSPQHPGATPPTGEPGFPVVHQDLSHIQQPAAVVAAL    2597
scSEN1      AESPSTSSGTKKKSSIFGGMSVP------SAVVPK---TFPDVD----SNKKAAAVVGKK    1995
                 .  * : *..**     *       .*. *.    ** *.       :: ****.

hsSETX      SS---------------------------------HKPPVRGEPPAASPEASTCQSKCDD    2624
scSEN1      KNNKHVCFSDDVSFIPRNDEPEIKVTRSLSSVLKEKQLGLKETRTISPPEISNNE--DDD    2053
            ..                                 ::  ::     : ** *. :   **
```

```
hsSETX      PEEELCHRREARAF----SEGEQEKCGSETHHTRRNSRWDKRTLEQEDSSS---------    2671
scSEN1      DEDDYTPSISDSSLMKSEANGRNNRVASHNQ-NFSASIYDDPQVSQAKQTQVPAAITKHR    2112
             *::     .  ::    ::*.::: .*..: .   * :*.  :.* ..:.

hsSETX      --------KKRKLL----------------------------------------------    2677
scSEN1      SSNSVLSGGSSRILTASDYGEPNQNGQNGANRTLSQHVGNANQYSTAPVGTGELHETLPA    2172
                     . ::*

hsSETX      -----------------------------------------------------------     2677
scSEN1      HPQDSYPAEAEDPYDLNPHPQPQSSAFKGPGSGPTGTRNSSRRNASSSPFIPKKRKPRS     2231
```

2-3: CLUSTAL O(1.2.4) multiple sequence alignment **[ALS8]**

```
hsVAPB          MAKVEQVLSLEPQHELKFRGPFTDVVTTNLKLGNPTDRNVCFKVKTTAPRRYCVRPNSGI    60
scSCS2          MSAV----EI-SPDVLVYKSPLTEQSTEYASISNNSDQTIAFKVKTTAPKFYCVRPNAAV    55
                *: *    .:   . * ::.*:*:  *   .:.* :*:.:.*****:***: ******:.:

hsVAPB          IDAGASINVSVMLQPFDYDPNE--KSKHKFMVQSMFAPTDT--SDMEAVWKEAKPEDL--    114
scSCS2          VAPGETIQVQVIFLGLTEEPAADFKCRDKFLVITLPSPYDLNGKAVADVWSDLEAEFKQQ    115
                :  * :*:*.*::  :  :*    *.:.**:* :: :* *   . :  **.: : *

hsVAPB          -MDSKLRCVFELPAENDKPHDVEINKIISTTASKTETPIVSKSLS----SSLDDTEVKKV    169
scSCS2          AISKKIKVKYLISPDVHPAQNQNIQE-----NKETVEPVVQDSEPKEVPAVVNEKEVPAE    170
                 :..*::  : :  : .  :: :*::      .:*  *:*..*      : :::.**

hsVAPB          MEECKRLQGEVQRLREENKQ-----FKEEDGLR-MRKTVQSNSPI--SALAPTGKEEGLS    221
scSCS2          P----ETQPPVQVKKEEVPPVVQKTVPHENEKQTSNSTPAPQNQIKEAATVPAENE-SSS    225
                     . *  **  :**        . .*:  :  ..*   :. *  :* .*: :* . *

hsVAPB          TRLLAL-VVLFFIVGVIIGKIAL       243
scSCS2          MGIFILVALLILVLGWFYR----       244
                  :: * .:*::::* :
```

CLUSTAL O(1.2.4) multiple sequence alignment **[ALS8]**

```
hsVAPB          MAKVEQVLSLEPQHELKFRGPFTDVVTTNLKLGNPTDRNVCFKVKTTAPRRYCVRPNSGI    60
scSCS22         -------MRI-VPEKLVFKAPLNKQSTEYIKLENDGEKRVIFKVRTSAPTKYCVRPNVAI    52
                       : :   .:* *:.*:..  *  :** *  ::.* ***:*:** :****** .*

hsVAPB          IDAGASINVSVMLQPFDYDP----NEKSKHKFMVQSMFAPTD-----TSDMEAVWKEAKP    111
scSCS22         IGAHESVNVQIVFLGLPKSTADDEMDQKRDKFLIVTLPIPAAYQNVEDGELLSDWPNLEE    112
                *.*  *:**.:::  :  .      ::.:.**:: ::  *:       .:: : * : :

hsVAPB          E---DLMDSKLRCVFELPAENDKPHDVEINKIISTTASKTETPIVSKSLSSSLDDTEVKK    168
scSCS22         QYKDDIVFKKIKI-FH--------------------------------------------    127
                :   *:: .*::  *.

hsVAPB          VMEECKRLQGEVQRLREENKQFKEEDGLRMRKTVQSNSPISAL-APTGKEEGLSTRLLA-    226
scSCS22         ---------SVL----------------PKRKPSGNHDAESARAPSAGNGQSLSSRALLI    162
                         . :                  **   .:.  **    :*: :.**:* *

hsVAPB          LVVLFFIVGVIIGKIAL    243
scSCS22         ITVIALLVGWIYY----    175
                :.*: ::** *
```

2-4: CLUSTAL O(1.2.4) multiple sequence alignment **[ALS11]**

```
hsFIG4      --------------MPT----AAAPIISSVQKLVLYETRARYFLVGSNNAETKYRVLKID     42
scFIG4      MNNDAMEHTLGGGILTTSGSKQRKTSKFVMGKYTLYETKDRMYIVGSNKRETMFRILEID     60
                           : *              : * .****: * ::****: ** :*:*:**

hsFIG4      RTEPKDLV-IIDDRHVYTQQEVRELLGRLDLGNRTKMGQKGSSGLFRAVSAFGVVGFVRF     101
scFIG4      LTVPRGELTVLEDNVFFTRNEIMNVLASLE--------EATEDGLHKKITGYGLLGFIKF     112
             * *:. : :::*. .:*::*: ::*. *:         :  ..**.: ::.:*::*::*

hsFIG4      LEGYYIVLITKRRKMADIGGHAIYKVEDTNMIYIPNDSVR-VTHPDEARYLRIFQNVDLS     160
scFIG4      TCWYYLIMVTKYSQVAVIGGHGIYHIDGIDIIPITNNYKKPEKSSDEARLLNIFKDLDLT     172
               **::::**  ::* ****.**:::. ::* * *:  :  .  **** *.**:::**:

hsFIG4      SNFYFSYSYDLSHSLQYNLTVLRMPLEMLKSEMTQNRQESFDIFEDEGLITQGGSGVFGI     220
scFIG4      KTFYFSYTYDITNTLQTNILREKLK--AV---------DRCDITIPC-----------GI     210
            ..*****:**::::** *:    ::     :          :  **              **

hsFIG4      CSEPYMKYVWNGELLDIIKST--VHRDWLLYIIHGFCGQSKLLIYGRPVYVTLIARRSSK     278
scFIG4      -TDYNEMFVWNNNLLSPIFACIDTVFDWFQCIIHGFIDQVNVSVLGKSIYITLIARRSHH     269
             ::    :***.:**. * :   .  **:  ***** .* :: : *: :*:******* :

hsFIG4      FAGTRFLKRGANCEGDVANEVETEQILCDASVMSF--------TAGSYSSYVQVRGSVPL     330
scFIG4      FAGARFLKRGVNNKGHVANEVETEQIVTDMILTPFHQPGNGFFDSDRYTSFVQHRGSIPL     329
            ***:******.* :*.**********: *  :  *          :. *:*:** ***:**

hsFIG4      YWSQDISTMMPKPPITLDQADPFAHVAALHFDQMFQRFGSP-IIILNLVKEREKRKHERI     389
scFIG4      YWTQDASNLTTKPPIRINVVDPFFSPAALHFDNLFQRYGGGTIQILNLIKTKEKTPRETK     389
            **:** *.:  **** :: .***   ******::***:*.  * ****:* :**  :*

hsFIG4      LSEELVAAVTYLNQFLPPEHTIVYIPWDMAKYTKSKLCNVLDRLNVIAESVVKKTGFFVN     449
scFIG4      LLWEFEQCIDYLNEFLPTLKKLDYTSWDMSRASKQDGQGVIEFLEKYAVNTVTTTGIFHN     449
            *  *:  .: ***:***  :.: *  ***:: :*..  .*:: *:  *  ..*..**:* *

hsFIG4      RPDSYCSILRPDEKWNELGGCVIPTGRLQTGILRTNCVDCLDRTNTAQFMVGKCALAYQL     509
scFIG4      GPDF-------------------ASTKIQEGICRSNCIDCLDRTNAAQFVIGKRALGCQL     490
             **                    : ::* ** *:**:*******:***::** **. **

hsFIG4      YSLGLIDKPNLQFDTDAVRLFEELYEDHGDTLSLQYGGSQLVHRVKTYRKIAPWTQHSKD     569
scFIG4      KSLGIIDNSYLEYDSDIVNILTELFHDLGDTIALQYGGSHLVNTMETYRKINQWSSHSRD     550
             ***:**:  *::*:* *.:: **:.* ***::******:**: ::*****  *:.**:*

hsFIG4      IMQTLSRYYSNAFSDADRQDSINLFLGVFHPTEGKPHLWELPTDFYLHHKNTMRLLPTRR     629
scFIG4      MIESIKRFYSNSFVDAQRQDAINLFLGHYSWREGFPSLWEMNTDFYLHNAYSL--NMPKR     608
            :::::.*:***:* **:***:****** :   ** * ***: ******:  ::     :*

hsFIG4      SYTYWWTPEVIKHLPLPYDEVICAVNL---KKLIVKKFHKYEEEI-DIHNEFFRPYELSS     685
scFIG4      SYIHWWNDYNIKSVKELINEELIATGNDVTREKIIKNVRGYPGAFDNYWNEYYLPRSVTW     668
            ** :**.    ** :     :* : *..     :: *:*:.: *    : :  **:: * .::

hsFIG4      FDDTFCLAMTSSARDFMPK-TVGIDPSPFTVRKPDETGKSVLG---NKS-N-REEAVLQR     739
scFIG4      IRDLFAYNMNSTRRYHNALSKQDKAMSPFTSRKQSWLNNKLKMITSSKSLEKAEGRVVET     728
            : * *.  *.*: *  .     . .   **** **  .  .:.:      .** :  *  *::

hsFIG4      KTAASAPPPPSEEAVS----SSSEDDSGTDREEEGSVSQRSTPVKMTDAGDSAKVTENVV     795
scFIG4      TDLDRDTSPKQELELYEHYLHIISDRSQKLEEKMNSFSYSKYPIFISH-------ESSEI     781
            .          * .*  :          .* * . .*: .*.*  . *: ::.         .. :

hsFIG4      QPMKELYGINLSDGLSEEDFSIYSRFVQLGQSQHQKDKNSQQPCSRCSDGVIKLTPISAF     855
scFIG4      PPMRKVIGEPLVDIA--------EDFTDVYDDDDDGDE----NDEMTTEALLIAPDHVS     829
             **::: *   * *           . *.:: :.:.. *.:      .. :  .: ::*  .
```

```
hsFIG4      SQDNIYEVQ------PPRVDRKSTEIFQAHIQASQGIMQPLGKEDSSMYREYIRNRYL--     907
scFIG4      VDEKFYEKVLNVDDYKPALDDYS-----AVI--------HIKPDNLQLYRDLCFSKDIQL     876
             ::::**          * :*  *      * *         :  :: .:**:   .: :

hsFIG4      ---  907
scFIG4      DFQ  879
```

2-5: CLUSTAL O(1.2.4) multiple sequence alignment **[ALS13]**

```
hsATXN2      MRSAAAAPRSPAVATESRRFAAARWPGWRSLQRPARRSGRGGGGAAPGPYPSAAPPPPGP    60
scPBP1       ------------------------------------------------------------    0

hsATXN2      GPPPSRQSSPPPSASDCFGSNGNGGGAFRPGSRRLLGLGGPPRPFVVVLLPLASPGAPPAA    120
scPBP1       ------------------------------------------------------------    0

hsATXN2      PTRASPLGARASPPRSGVSLARPAPGCPRPACEPVYGPLTMSLKPQQQQQQQQQQQQQQQ    180
scPBP1       ------------------------------------------------------------    0

hsATXN2      QQQQQQQPPPAAANVRKPGGSGLLASPAAAPSPSSSSVSSSSATAPSSVVAATSGGGRPG    240
scPBP1       ----------MKGNFRKRD---------------------------SS-TNSRKGG----    18
                         .*.** .                           ** . : .**

hsATXN2      LGRGRNSNKGLPQSTISFDGIYA--------NMRMVHILTSVVGSKCEVQVKNGGIYEGV    292
scPBP1       NSDSNYTNGGVPNQNNS-SMFYENPEITRNFDDRQDYLLANSIGSDVTVTVTSGVKYTGL    77
              . .. :* *:*:.. * . :*         : *  ::*:. :**.  * *..*  * *:

hsATXN2      FKTYSPKCD----LVLDAAH--EKSTESSSGPKREEIMESILFKCSDFVVVQFKDMDSSY    346
scPBP1       LVSCNLESTNGIDVVLRFPRVADSGVSDSVDDLAKTLGETLLIHGEDVAELELKNIDLSL    137
             : : . :.     :**   :  :.....* .   : : *::*:: .*.. :::*::* *

hsATXN2      AKR----------------------------DAFTDSAISAKVNGEHKEKDLEPWDAGEL    378
scPBP1       DEKWENSKAQETTPARTNIEKERVNGESNEVTKFRTDVDISGSGREIKERKLEKWTPEEG    197
              ::                              *  ..  :  . * **:.** *   *

hsATXN2      TANEELE--ALENDVSNGWDPNDMFRYNEENYGVVSTYDSSLSSYTVPLERDNSEEFLKR    436
scPBP1       AEHFDINKGKALEDDSASW---DQFAVNEKKFGVKSTFDEHLYTTKINKDDP---NYSKR    251
             : : :::     :* * .*   * *  **:::** **:*. * : .:  :     :: **

hsATXN2      EARANQLAEEIESSAQYKA-------RVALENDDRSEEEKYTAVQRNSSERE------GH    483
scPBP1       LQEAERIAKEIESQGTSGNIHIAEDRGIIIDDSGLDEEDLYSGVDRRGDELLAALKSNSK    311
               .*:::*:****..            : :::.. .**: *:.*:*...*        .:

hsATXN2      SINTRENKYIPPGQRNREVISWGSGRQNSPRMGQPGSGSMPSRSTSHTSDFNPNSGSDQR    543
scPBP1       PNSNKGNRYVPPTLRQQPHH-----------M-D------PAIISSS------NSNKNEN    347
               ..: *:*:**  *::              * :      *:  :*       **..::.

hsATXN2      VVNGGVPWPSPCPSPSSRPPSRYQSGPNSLPPRAATPTRPPSRPPSRPSRPPSHPSAHGS    603
scPBP1       AVSTDTSTPAAAGAPEGKPPQKTSKNKKSLS-----------------------------    378
             .*. ..  *: . :*..:**.: ... :**

hsATXN2      PAPVSTMPKRMSSEGPPRMSPKAQRHPRNHRVSAGRGSISSGLEFVSHNPPSEAATPPVA    663
scPBP1       ---------------------------------SKEAQIEELKKFSEK------FKVP--    397
                                              : ...*..  :* .:       . *

hsATXN2      RTSPSGGTWSSVVSGVPRLSPKTHRPRSPRQNSIGNTPSGPVLASPQAGIIPTEAVAMPI    723
scPBP1       -------------YDIPKDMLEVLKRSSSTLKSNSSLPPKPISKTPSAK-----------    433
                           .:*:   :. :  *   :* .. *  *:  :*.*

hsATXN2      PAASPTPASPASNRAVTPSSEAKDSRLQDQRQNS--PAGNKENIKPNETSPSFSKAENKG    781
scPBP1       --------------TVSPTTQISAGKSESRRSGSNISQGQSSTGHTTRSSTSLRRR-NHG    478
                           :*:*::: . .: :.:*..*    *:... : ..:* *: :  *:*

hsATXN2      ISPVVSEHRKQIDDLK--KFKNDFRLQPSSTSESMDQLLNKNREGEKSRDLIKDKIEPSA    839
scPBP1       --SFFGAKNPHTNDAKRVLFGKSFNMFIKSKE-A----HDEKKKGDD----ASENMEP--    525
                ... :. : :* *   * :.*.:  .*.. :     :::::*:.     .:::**
```

```
hsATXN2      KDSFIENSSSNCTSGSSKPNSPSISPSILSNTEHKRGPEVTSQGVQTSSPACKQEKDDKE    899
scPBP1       --FFIEKPYF-------------TAPTWLNTIEESY---------KTFFP----DED--T    555
                ***:                 :*: *.. *..          :*  *    ::*

hsATXN2      EKKDAAEQVRKSTLNP---NAKEFNPRSFSQPKPSTTPTSPRPQAQPSPSMVGHQQP---    953
scPBP1       AIQEAQTRFQQRQLNSMGNAVPGMNPAMGMNMGGMMGFPMGGPSASPNPMMNGFAAGSMG    615
               ::*  :.::  **     .  :**    :           *.*.*.* * *.

hsATXN2      --TPVYTQPVCFAPNMMYPVPVSPGVQPLYPIPMTPMPVNQAKT--YRAVPNMPQQRQDQ    1009
scPBP1       MYMPFQPQPMFYHPSMPQMMPVM--------------GSNGAEEGGGNISPHVP------    655
                *.  **: : *.*   :**                 * *:    .  *::*

hsATXN2      HHQSAMMHPASAAGPPIAATPPAYST-----QYVAYSPQQFPNQPLVQ----HVPHYQ--    1058
scPBP1       ---AGFMA--AGPGAPMGAF--GYPGGIPFQGMMGSGPSGMPANGSAMHSHGHSRNYHQT    708
                :.:*   :. * *:.*   .*         :. .*. :* :  .     *  :*:

hsATXN2      SQHPHVYSPVIQGNARMMAPPTHAQPGLVSSSATQYGAHEQTHAMYACPKLPYNKETSPS    1118
scPBP1       SHHGHHNSS-TSGHK---------------------------------------------    722
             *:* *  *   .*:

hsATXN2      FYFAISTGSLAQQYAHPNATLHPHTPHPQPSATPTGQQQSQHGGSHPAPSPVQHHQHQAA    1178
scPBP1       ------------------------------------------------------------    722

hsATXN2      QALHLASPQQQSAIYHAGLAPTPPSMTPASNTQSPQNSFPAAQQTVFTIHPSHVQPAYTN    1238
scPBP1       ------------------------------------------------------------    722

hsATXN2      PPHMAHVPQAHVQSGMVPSHPTAHAPMMLMTTQPPGGPQAALAQSALQPIPVSTTAHFPY    1298
scPBP1       ------------------------------------------------------------    722

hsATXN2      MTHPSVQAHHQQQL      1312
scPBP1       --------------      722
```

2-6: CLUSTAL O(1.2.4) multiple sequence alignment **[ALS14; FTD-ALS6]**

```
hsVCP           ---------MASGADSK-GDDLSTAILKQKNRPNRLIVDEAINEDNSVVSLSQPKMDELQ   50
scCDC48         MGEEHKPLLDASGVDPREEDKTATAILRRKKKDNMLLVDDAINDDNSVIAINSNTMDKLE   60
                         ***.* :  *. :****::*:: * *:**:***:****:::.. .**:*:

hsVCP           LFRGDTVLLKGKKRREAVCIVLSDDTCSDEKIRMNRVVRNNLRVRLGDVISIQPCPDVKY   110
scCDC48         LFRGDTVLVKGKKRKDTVLIVLIDDELEDGACRINRVVRNNLRIRLGDLVTIHPCPDIKY   120
                ********:*****:::* *** **   .*    *:*******:****:::*:****:**

hsVCP           GKRIHVLPIDDTVEGITGNLFEVYLKPYFLEAYRPIRKGDIFLVRGGMRAVEFKVVETDP   170
scCDC48         ATRISVLPIADTIEGITGNLFDVFLKPYFVEAYRPVRKGDHFVVRGGMRQVEFKVVDVEP   180
                ..** **** **:********:*:*****:*****:**** *:****** ******:.:*

hsVCP           SPYCIVAPDTVIHCEGEPIKREDEEESLNEVGYDDIGGCRKQLAQIKEMVELPLRHPALF   230
scCDC48         EEYAVVAQDTIIHWEGEPINREDEENNMNEVGYDDIGGCRKQMAQIREMVELPLRHPQLF   240
                . *.:** **:** *****:*****:.:*************:***:********* **

hsVCP           KAIGVKPPRGILLYGPPGTGKTLIARAVANETGAFFFLINGPEIMSKLAGESESNLRKAF   290
scCDC48         KAIGIKPPRGVLMYGPPGTGKTLMARAVANETGAFFFLINGPEVMSKMAGESESNLRKAF   300
                ****:*****:*:**********:*****************:***:************

hsVCP           EEAEKNAPAIIFIDELDAIAPKREKTHGEVERRIVSQLLTLMDGLKQRAHVIVMAATNRP   350
scCDC48         EEAEKNAPAIIFIDEIDSIAPKRDKTNGEVERRVVSQLLTLMDGMKARSNVVVIAATNRP   360
                ***************:*:*****:**:******:**********:* *::*:*:******

hsVCP           NSIDPALRRFGRFDREVDIGIPDATGRLEILQIHTKNMKLADDVDLEQVANETHGHVGAD   410
scCDC48         NSIDPALRRFGRFDREVDIGIPDATGRLEVLRIHTKNMKLADDVDLEALAAETHGYVGAD   420
                *****************************:*:*************** :* ****:****

hsVCP           LAALCSEAALQAIRKKMDLIDLEDETIDAEVMNSLAVTMDDFRWALSQSNPSALRETVVE   470
scCDC48         IASLCSEAAMQQIREKMDLIDLDEDEIDAEVLDSLGVTMDNFRFALGNSNPSALRETVVE   480
                :*:******:* **:*******:::  *****::**.****:**:**.:************

hsVCP           VPQVTWEDIGGLEDVKRELQELVQYPVEHPDKFLKFGMTPSKGVLFYGPPGCGKTLLAKA   530
scCDC48         SVNVTWDDVGGLDEIKEELKETVEYPVLHPDQYTKFGLSPSKGVLFYGPPGTGKTLLAKA   540
                  :***:*:***:::*.**:* *:*** ***::  ***::************ ********

hsVCP           IANECQANFISIKGPELLTMWFGESEANVREIFDKARQAAPCVLFFDELDSIAKARGGNI   590
scCDC48         VATEVSANFISVKGPELLSMWYGESESNIRDIFDKARAAAPTVVFLDELDSIAKARGGSL   600
                :*.* .*****:******:**:****:*:*:****** *** *:*:************.:

hsVCP           GDGGGAADRVINQILTEMDGMSTKKNVFIIGATNRPDIIDPAILRPGRLDQLIYIPLPDE   650
scCDC48         GDAGGASDRVVNQLLTEMDGMNAKKNVFVIGATNRPDQIDPAILRPGRLDQLIYVPLPDE   660
                **.***:***:**:*******.:*****:******** ****************:*****

hsVCP           KSRVAILKANLRKSPVAKDVDLEFLAKMTNGFSGADLTEICQRACKLAIRESIESEIRRE   710
scCDC48         NARLSILNAQLRKTPLEPGLELTAIAKATQGFSGADLLYIVQRAAKYAIKDSIEAHRQHE   720
                ::*::**:*:***:*:   .::*  :** *:*******  * ***.* **::***:. ::*

hsVCP           RERQTNP------------SAMEVEEDDPVPEIRRDHFEEAMRFARRSVSDNDIRKYEMF   758
scCDC48         AEKEVKVEGEDVEMTDEGAKAEQEPEVDPVPYITKEHFAEAMKTAKRSVSDAELRRYEAY   780
                 *::.:              .* :  * **** * ::** ***: *:***** ::*:** :

hsVCP           AQTLQQSRG-FGSFRFPSGNQGGAGPSQG-----SGGGTGGS-VYTEDNDDDLYG        806
scCDC48         SQQMKASRGQFSNFNFNDAPLGTTATDNANSNNSAPSGAGAAFGSNAEEDDDLYS        835
                :* :: *** *..*.* ..  * :. .:.      : .*:*.:   . ::*****.
```

2-7: CLUSTAL O(1.2.4) multiple sequence alignment **[ALS15]**

```
hsUBQLN2      MAENGESSGPPRPSRGPAAAQGSAAAPAEPKIIKVTVKTPKEKEEFAVPENSSVQQFKEA   60
scDSK2        ------------------------------MSLNIHIKSGQDKWEVNVAPESTVLQFKEA   30
                                              ::: :*: ::* *. *  :*:* *****

hsUBQLN2      ISKRFKSQTDQLVLIFAGKILKDQDTLIQHGIHDGLTVHLVIKSQNRPQGQSTQPSNAAG   120
scDSK2        INKANGIPVANQRLIYSGKILKDDQTVESYHIQDGHSVHLVKSQ-PKPQTASAAGANNAT   89
              *.*     . :  **::******::*: .: *:** :**** ..  :**  *:  :* *

hsUBQLN2      TNTTSASTPRSNSTPISTNSNPFGLGSLGGLAGLSSLGL---SSTNFSELQSQMQQQLMA   177
scDSK2        ATG---AAAGTGATPNMSSGQSAGFNPLADLTSARYAGYLNMPSADMFGPDGGALNNDSN   146
              :.    ::  :.:**  :..:  *:. *..*:.    *     *:::   :.   ::

hsUBQLN2      SPEMMIQIMENPFVQ----SMLSNPDLMRQLIMANPQMQQLI------QRNPEISHLLNN   227
scDSK2        NQDELLRMMENPIFQSQMNEMLSNPQMLDFMIQSNPQLQAMGPQARQMLQSPMFRQMLTN   206
              . : ::::****:.*    .*****:::  :* :***:* :        :.* : ::*.*

hsUBQLN2      PDIMRQTLEIARNPAMMQEMMRNQDLALSNLESIPGGYNALRRMYTDIQEPMLNAAQEQF   287
scDSK2        PDMIRQSMQFARMMDPNAGMG-------------------------------------SA   229
              **::**::::**       *                                      .

hsUBQLN2      GGNPFASVGSSSSSGEGTQPSRTENRDPLPNPWAPPPATQSSATTSTTTSTGSGSGNSSS   347
scDSK2        GGA----------------------ASAFPAPGGDAPEEGSNTNTTSSSNTGNNAGTNAG   267
              **                        . :* * .  *   *.:.*::::.**..:*..:.

hsUBQLN2      NATGNTVAAANYVASI-------FSTPGMQSLLQQITENPQLIQNMLSAPYMRSMMQSLS   400
scDSK2        TNAGANTAANPF-ASLLNPALNPFANAGNAASTGMPAFDPALLASMFQPPVQA-------   319
              . :* ..**  : **:       *:. *  :     : :* *: .*:. *

hsUBQLN2      QNPDLAAQMMLNSPLFTANPQLQEQMRPQLPAFLQQMQNPDTLSAMSNPRAMQALMQIQQ   460
scDSK2        ------------------------------------------------------------   319


hsUBQLN2      GLQTLATEAPGLIPSFTPGVGVGVLGTAIGPVGPVTPIGPIGPIVPFTPIGPIGPIGPTG   520
scDSK2        ------------------------------------------------------------   319


hsUBQLN2      PAAPPGSTGSGGPTGPTVSSAAPSETTSPTSESGPNQQFIQQMVQALAGANAPQLPNPEV   580
scDSK2        -------------------------------------------------SQAEDTRPPEE   330
                                                               ::* :   **

hsUBQLN2      RFQQQLEQLNAMGFLNREANLQALIATGGDINAAIERLLGSQPS  624
scDSK2        RYEHQLRQLNDMGFFDFDRNVAALRRSGGSVQGALDSLLNGDV-  373
              *:::**.*** ***:: : *: **  :**.::.*:: **..:
```

2-8: CLUSTAL O(1.2.4) multiple sequence alignment **[ALS16]**

```
hsSIGMAR1       MQWAVGRRWAWAALLLAVAAV-LTQVVWLWLGTQSFVFQREEIAQLARQYAG---LDHEL 56
scERG2          ------MKFFPLLLLIGVVGYIMNVLFTTWLPT-NYMFDPKTLNEICNSVISKHNAAEGL 53
                       ::    **:.*..  :. :.  ** * .::*: : : ::...  .     . *

hsSIGMAR1       AFSRLIVELRRLHPG-----HVLPDEELQWVFVNAGGWMGAMCLLHASLSEYVLLFGTAL 111
scERG2          STEDLLQDVRDALASHYGDEYINRYVKEEWVFNNAGGAMGQMIILHASVSEYLILFGTAV 113
                : . *: ::*    .     ::    : :*** **** ** * :****:***::*****:

hsSIGMAR1       GSRGHSGRYWAEISDTIISGTFHQWREGTTKSEVFYPGETVVHGPGEATAVEWGPNTWMV 171
scERG2          GTEGHTGVHFADDYFTILHGTQIAALPYATEAEVYTPGMTHHLKKGYAKQYSMPGGSFAL 173
                *:.**:* ::*:   **: **       :*::**: ** *     * *.  .   .:: :

hsSIGMAR1       EYGRGVIPSTLAFALADTVFSTQDFLTLFYTLRSYARGLRLELTT-YLFGQDP        223
scERG2          ELAQGWIPCMLPFGFLDTFSSTLDLYTLYRTVYLTARDMGKNLLQNKKF----        222
                * .:* **. * *.: **. ** *: **: *:   **.:  :*     *
```

2-9: CLUSTAL O(1.2.4) multiple sequence alignment **[ALS17/ALS-FTD7]**

```
hsCHMP2B        ---MASLFKK-KTVDDVIKEQNRELRGTQRAIIRDRAALEKQEKQLELEIKKMAKIGNKE  56
scDID4          MSLFEWVFGKNVTPQERLKKNQRALERTQRELEREKRKLELQDKKLVSEIKKSAKNGQVA  60
                   :  :* *  * :: :*:::* *. *** : *::  ** *:*:*  **** ** *:

hsCHMP2B        ACKVLAKQLVHLRKQKTRTFAVSSKVTSMSTQTKVMNSQMKMAGAMSTTAKTMQAVNKKM  116
scDID4          AAKVQAKDLVRTRNYIQKFDNMKAQLQAISLRIQAVRSSDQMTRSMSEATGLLAGMNRTM  120
                *.** **:**: *:   :   :.::: ::* : :.:.*. :*: :** ::  : .:*:.*

hsCHMP2B        DPQKTLQTMQNFQKENMKMEMTEEMINDTLDDDIFD-GSDDEEESQDIVNQVLDEIGIEIS  175
scDID4          NLPQLQRISMEFEKQSDLMGQRQEFMDEAIDNVMGDEVDEDEEADEIVNKVLDEIGVDLN  180
                :  :  :   :*:*:.  *   :*::::::*:::.   *::**:::***:******:::.

hsCHMP2B        GKMAKAPSAARSLPSASTSKATI-------------SDEEIERQLKALGVD-  213
scDID4          SQLQSTPQNLVSNAPIAETAMGIPEPIGAGSEFHGNPDDDLQARLNTLKKQT  232
                .:: .:*.   *    : :   *              *:::: :*::*  :
```

2-10: CLUSTAL O(1.2.4) multiple sequence alignment **[ALS18]**

```
hsPFN1      MAGWNAYIDNLMADGTCQDAAIVGYKDSPSVWAAVPGKTFVNITPAEVGVLVGK--DRSS    58
scPFY1      -MSWQAYTDNLIGTGKVDKAVIY-SRAGDAVWATSGGLS---LQPNEIGEIVQGFDNPAG    55
              .*:** ***:. *. :.*.*   : . :***:  * :   : * *:* :*    : :.

hsPFN1      FYVNGLTLGGQKCSVIRDSLLQDGEFSMDLRTKSTGGAPTFNVTVTKTDKTLVLLMGKEG    118
scPFY1      LQSNGLHIQGQKFMLLRADDR-----SIYGRHDA------EGVVCVRTKQTVIIAHYPPT    104
            :  *** : ***  ::* .        *:  * .:        .*. .:*.:*:::

hsPFN1      VHGGLINKKCYEMASHLRRSQY        140
scPFY1      VQAGEATKIVEQLADYLIGVQY        126
            *:.*  .*   ::*.:*   **
```

2-11: CLUSTAL O(1.2.4) multiple sequence alignment **[ALS20]**

```
hsHNRNPA1      ------------------------------------------------------------ 0
scHRP1         MSSDEEDFNDIYGDDKPTTTEEVKKEEEQNKAGSGTSQLDQLAALQALSSSLNKLNNPNS 60


hsHNRNPA1      ------------------------------------------------------------ 0
scHRP1         NNSSSNNSNQDTSSSKQDGTANDKEGSNEDTKNEKKQESATSANANANASSAGPSGLPWE 120


hsHNRNPA1      -------------------------MSKSESPKEPEQLRKLFIGGLSFETTDESLRSHFE 35
scHRP1         QLQQTMSQFQQPSSQSPPQQQVTQTKEERSKADLSKESCKMFIGGLNWDTTEDNLREYFG 180
                                         .: .. .  ::  *:*****.::**::.**.:*

hsHNRNPA1      QWGTLTDCVVMRDPNTKRSRGFGFVTYATVEEVDAAMNARPHKVDGRVVEPKRAVSREDS 95
scHRP1         KYGTVTDLKIMKDPATGRSRGFGFLSFEKPSSVDEVV-KTQHILDGKVIDPKRAIPRDEQ 239
               ::**:**  :*:** * *******::: . ..** .:    * :**:*::****: *::.

hsHNRNPA1      QRPGAHLTVKKIFVGGIKEDTEEHHLRDYFEQYGKIEVIEIMTDRGSGKKRGFAFVTFDD 155
scHRP1         DKT------GKIFVGGIGPDVRPKEFEEFFSQWGTIIDAQLMLDKDTGQSRGFGFVTYDS 293
               ::        *******  *.. :.:.::*.*:*.*   ::* *:.:*:.***.***:*.

hsHNRNPA1      HDSVDKIVIQKYHTVNGHNCEVRKALSKQEMASASSSQRGRSGSGNFGGGRGGGFGGNDN 215
scHRP1         ADAVDRVCQNKFIDFKDRKIEIKRAEPRHMQ-QKSSNN-------------GGNNGGNNM 339
                *:**::  :*:  .:.:: *:::*  ::   . **.:             **. ***:

hsHNRNPA1      FGRGGNFSGRGGFGGSRGGGGYGGSGDG----------------YNGFGNDGG------Y 253
scHRP1         NRRGGNFGNQGDFNQMYQNPMMGGYNPMMNPQAMTDYYQKMQEYYQQMQKQTGMDYTQMY 399
                 *****..:*.*.    .   ** .                  *: : :: *      *

hsHNRNPA1      G-------GGGPGYSGGSRGY-------------------------GSGGQGYGNQGSGY 281
scHRP1         QQQMQQMAMMMPGFAMPPNAMTLNQPQQDSNATQGSPAPSDSDNNKSNDVQTIGNTSNTD 459
                          **::   ..                          ... *  ** ..

hsHNRNPA1      GGSGS---------YDSYNNGGGGGFGGGSGSNFGGGGSYNDFGN--YNNQSSNFGPMKG 330
scHRP1         SGSPPLNLPNGPKGPSQYNDDHNSGYGYN-----------RDRGDRDRNDRDRDYNHRSG 508
               .**            ..**:. ..*:* .           .* *:   *::. ::.  .*

hsHNRNPA1      GNFGGRSSGPYGGGGQYFAKPRNQGGYGGSSSSSSYGSGRRF   372
scHRP1         GNHRRNG---RGGRGGYNRRN---NGYHP------YNR----   534
               **.  ..    ** * *  :    .**        *.
```

2-12: CLUSTAL O(1.2.4) multiple sequence alignment **[ALS22]**

```
hsTUBA4A      MRECISVHVGQAGVQMGNACWELYCLEHGIQPDGQMPSDK-TIGGGDDSFTTFFCETGAG   59
scTUB1        MREVISINVGQAGCQIGNACWELYSLEHGIKPDGHLEDGLSKPKGGEEGFSTFFHETGYG   60
              *** **::***** *:********.*****:***:: ..  .  **::.*:*** *** *

hsTUBA4A      KHVPRAVFVDLEPTVIDEIRNGPYRQLFHPEQLITGKEDAANNYARGHYTIGKEIIDPVL   119
scTUB1        KFVPRAIYVDLEPNVIDEVRNGPYKDLFHPEQLISGKEDAANNYARGHYTVGREILGDVL   120
              *.****::*****.****:*****::********:***************:*:**:. **

hsTUBA4A      DRIRKLSDQCTGLQGFLVFHSFGGGTGSGFTSLLMERLSVDYGKKSKLEFSIYPAPQVST   179
scTUB1        DRIRKLADQCDGLQGFLFTHSLGGGTGSGLGSLLLEELSAEYGKKSKLEFAVYPAPQVST   180
              ******:*** ******. **:*******: ***:*.**.:*********::********

hsTUBA4A      AVVEPYNSILTTHTTLEHSDCAFMVDNEAIYDICRRNLDIERPTYTNLNRLISQIVSSIT   239
scTUB1        SVVEPYNTVLTTHTTLEHADCTFMVDNEAIYDMCKRNLDIPRPSFANLNNLIAQVVSSVT   240
              :******::*********:**:**********:*:***** **:::***.**:*:***:*

hsTUBA4A      ASLRFDGALNVDLTEFQTNLVPYPRIHFPLATYAPVISAEKAYHEQLSVAEITNACFEPA   299
scTUB1        ASLRFDGSLNVDLNEFQTNLVPYPRIHFPLVSYSPVLSKSKAFHESNSVSEITNACFEPG   300
              *******:*****.****************.:*:**:* .**:**. **:*********.

hsTUBA4A      NQMVKCDPRHGKYMACCLLYRGDVVPKDVNAAIAAIKTKRSIQFVDWCPTGFKVGINYQP   359
scTUB1        NQMVKCDPRDGKYMATCLLYRGDVVTRDVQRAVEQVKNKKTVQLVDWCPTGFKIGICYEP   360
              *********.***** ********* :**: *:  :*.*:::*:*********:** *:*

hsTUBA4A      PTVVPGGDLAKVQRAVCMLSNTTAIAEAWARLDHKFDLMYAKRAFVHWYVGEGMEEGEFS   419
scTUB1        PTATPNSQLATVDRAVCMLSNTTSIAEAWKRIDRKFDLMYAKRAFVHWYVGEGMEEGEFT   420
              **..*..:**.*:**********:***** *:*:*************************:

hsTUBA4A      EAREDMAALEKDYEEVGIDSYEDEDEGEE        448
scTUB1        EAREDLAALERDYIEVGADSYAEEEEF--        447
              *****:****:** *** *** :*:*
```

2-12 (continued): CLUSTAL O(1.2.4) multiple sequence alignment **[ALS22]**

```
hsTUBA4A      MRECISVHVGQAGVQMGNACWELYCLEHGIQPDGQMPSDK-TIGGGDDSFTTFFCETGAG   59
scTUB3        MREVISINVGQAGCQIGNACWELYSLEHGIKEDGHLEDGLSKPKGGEEGFSTFFHETGYG   60
              *** **::***** *:********.*****: **:: ..  .  **::.*:*** *** *

hsTUBA4A      KHVPRAVFVDLEPTVIDEIRNGPYRQLFHPEQLITGKEDAANNYARGHYTIGKEIIDPVL   119
scTUB3        KFVPRAIYVDLEPNVIDEVRTGRFKELFHPEQLINGKEDAANNYARGHYTVGREIVDEVE   120
              *.****::*****.****:*.* :::********.***************:*:**:* * 

hsTUBA4A      DRIRKLSDQCTGLQGFLVFHSFGGGTGSGFTSLLMERLSVDYGKKSKLEFSIYPAPQVST   179
scTUB3        ERIRKMADQCDGLQGFLFTHSLGGGTGSGLGSLLLENLSYEYGKKSKLEFAVYPAPQLST   180
              :****::*** ******. **:*******: ***:*.** :*********::*****:**

hsTUBA4A      AVVEPYNSILTTHTTLEHSDCAFMVDNEAIYDICRRNLDIERPTYTNLNRLISQIVSSIT   239
scTUB3        SVVEPYNTVLTTHTTLEHADCTFMVDNEAIYDICKRNLGISRPSFSNLNGLIAQVISSVT   240
              :******::*********:**:************:***.*.**:::*** **:*::**:*

hsTUBA4A      ASLRFDGALNVDLTEFQTNLVPYPRIHFPLATYAPVISAEKAYHEQLSVAEITNACFEPA   299
scTUB3        ASLRFDGSLNVDLNEFQTNLVPYPRIHFPLVSYAPILSKKRATHESNSVSEITNACFEPG   300
              *******:*****.****************.:***::* ::* **. **:*********.

hsTUBA4A      NQMVKCDPRHGKYMACCLLYRGDVVPKDVNAAIAAIKTKRSIQFVDWCPTGFKVGINYQP   359
scTUB3        NQMVKCDPTKGKYMANCLLYRGDVVTRDVQRAVEQVKNKKTVQMVDWCPTGFKIGICYEP   360
              ******** :***** ********* :**: *:  :*.*:::*:*********:** *:*

hsTUBA4A      PTVVPGGDLAKVQRAVCMLSNTTAIAEAWARLDHKFDLMYAKRAFVHWYVGEGMEEGEFS   419
scTUB3        PSVIPSSELANVDRAVCMLSNTTAIADAWKRIDQKFDLMYAKRAFVHWYVGEGMEEGEFT   420
              *:*:*..:**:*:*************:** *:*:*************************:

hsTUBA4A      EAREDMAALEKDYEEVGIDSYEDEDEGEE        448
scTUB3        EAREDLAALERDYIEVGADSYAEEF----        445
              *****:****:** *** *** :*
```

2-13: CLUSTAL O(1.2.4) multiple sequence alignment **[ALS25]**

```
hsKIF5A      ------------------MAETNNECSIKVLCRFRPLNQAEIL----RGDKF--IPIFQG   36
scSMY1       MHWNIISKEQSSSSVSLPTLDSSEPCHIEVILRAIPEKGLQNNESTFKIDPYENTVLFRT   60
                                 ::.: * *:*: *  * :  :      : * :    :*:

hsKIF5A      D----DSVVIGGKPYVFDRVFPPNTTQEQVYHACAMQIVKDVLAGYNGTIFAYGQTSSGK   92
scSMY1       NNPLHETTKETHSTFQFDKVFDANATQEDVQKFLVHPIINDVLNGYNGTVITYGPSFSGK   120
             :    ::.    . : **:**  *:***:* :  .  *::*** *****:::** : ***

hsKIF5A      THTMEGKLHDPQLMGIIPRIARDIFNHIYSMDE--NLEFHIKVSYFEIYLDKIRDLLDVT   150
scSMY1       SYSLIGSK---ESEGILPNICKTLFDTLEKNEETKGDSFSVSVLAFEIYMEKTYDLLVPL   177
             :::: *.    :  **:*.*.: :*: : . :*  . .* :.*  ****::*  ***

hsKIF5A      KTN--LSVHEDKNR--VPFVKGCTERFVSSPEEILDVIDEGKSNRHVAVTNMNEHSSRSH   206
scSMY1       PERKPLKLHRSSSKMDLEIKDICPAH-VGSYEDLRSYIQAVQNVGNRMACGDKTERSRSH   236
               .  *.:*....:  : : . *  : *.* *:: . *:  :.  :  . . : . ***

hsKIF5A      SIFLINIKQENMETEQKLSGKLYLVDLAGSEKVSKTGAEGAVLDEAKNINKSLSALGNVI   266
scSMY1       LVFQLHVEQRNRKDDILKNSSLYLVDLHGAEKFDKRTESTLSQDALKKLNQSIEALKNTV   296
              :* ::::*.* : :   ...***** *:**..*   .    *  *::*:*:.** *.:

hsKIF5A      SALA---------EGTKSYVPYRDSKMTRILQDSLGGNCRTTMFICCSPSSYNDAETKST   317
scSMY1       RSLSMKERDSAYSAKGSHSSAYRESQLTEVLKDSLGGNRKTKVILTCFLSNVP--TTLST   354
              :*:            .    **:*::*.:*:****** :*.::: *  *.     * **

hsKIF5A      LMFGQRAKTIKNTAS-----VNLELTAEQWKKKYEKEKEKTKAQKETIAKLEAELSRWR-   371
scSMY1       LEFGDSIRQINNKVTDNTTGLNLKKKMDLFIQDMKIKDDNYV---AQINILKAEIDSLKS   411
             * **:  : *:*..:     :**: . : : :. : :.::       *  *:**:.  :

hsKIF5A      -NGENVPETERLAGEEAALGAELCEETPVNDNSSIVVRIAPEERQKYEEE---IRRLYKQ   427
scSMY1       LHNKSLPEDDE--------------------KKMLEN-TKKENIKLKLQLDSITQLLSS   449
              :.:.:** :.                    ..:: . : :*. * : :   * :* ..

hsKIF5A      LD--DKDDEINQQ-SQLIEKLKQQMLDQEELLVSTRGDNEKVQRELSHLQSENDAAKDEV   484
scSMY1       STNEDPNNRIDEEVSEILTKRCEQIAQLELSFDRQMNSNSKLQQELEYKKSKEEA-----   504
                 * ::.*::: *::: *  :*: : *  :    ..*.*:*:**.: :*:::*

hsKIF5A      KEVLQALEELAVNYDQ----KSQEVEEKSQQNQLLVDELSQKV-------ATMLSLESEL   533
scSMY1       ------LESMNVRLLEQIQLQEREIQELLTTNAILKGELETHTKLTETRSERIKSLESSV   558
                   **.: *.  :    :.:*::*    * :* .**. :.          : ****.:

hsKIF5A      QRLQEVSGHQRKRIAEVLNGLMKDLSEFSVIVGNGEIKLPVEISGAIEEEFTVARLYISK   593
scSMY1       KELSL----NKSAIPSPRRGSMSS--------SSGNTMLHIEEGSEISNSP-----WSAN   601
             :.*.     ::. * .  .* *..        ..*:  * :* .. *.:.      : ::

hsKIF5A      IKSEVKSVVKRCRQLENLQVECHRKMEVTGRELSSCQLLISQHEAKIRSLTEYMQSVELK   653
scSMY1       --TSSKPLVWGARKVSSSS------IATTGSQES----F------VAR---PFKKGL---   637
               :. * :*  .*::.. .      : .** : *    :        *    : :.:

hsKIF5A      KRHLEESYDSLSDELAKLQAQETVHEVALKDKEPDTQDADEVKKALELQMESHREAHHRQ   713
scSMY1       ----------------------NLHSIKVTSSTPKSPSSGS-------------------   656
                                   .:*.: :... *.: .:..

hsKIF5A      LARLRDEINEKQKTIDELKDLNQKLQLELEKLQADYEKLKSEEHEKSTKLQELTFLYERH   773
scSMY1       ------------------------------------------------------------   656

hsKIF5A      EQSKQDLKGLEETVARELQTLHNLRKLFVQDVTTRVKKSAEMEPEDSGGIHSQKQKISFL   833
scSMY1       ------------------------------------------------------------   656
```

```
hsKIF5A      ENNLEQLTKVHKQLVRDNADLRCELPKLEKRLRATAERVKALEGALKEAKEGAMKDKRRY   893
scSMY1       ------------------------------------------------------------   656

hsKIF5A      QQEVDRIKEAVRYKSSGKRAHSAQIAKPVRPGHYPASSPTNPYGTRSPECISYTNSLFQN   953
scSMY1       ------------------------------------------------------------   656

hsKIF5A      YQNLYLQATPSSTSDMYFANSCTSSGATSSGGPLASYQKANMDNGNATDINDNRSDLPCG   1013
scSMY1       ------------------------------------------------------------   656

hsKIF5A      YEAEDQAKLFPLHQETAAS  1032
scSMY1       -------------------  656
```

2-14: CLUSTAL O(1.2.4) multiple sequence alignment **[ALS26]**

```
hsTIA1      ------------------------------------------------------------    0
scPUB1      MSENNEEQHQQQQQQQPVAVETPSAVEAPASADPSSEQSVAVEGNSEQAEDNQGENDPSV    60


hsTIA1      --------MEDEMPKTLYVGNLSRDVTEALILQLFSQIGPCKNCKMIMDTA-GNDPYCFV    51
scPUB1      VPANAITGGRETSDRVLYVGNLDKAITEDILKQYFQVGGPIANIKIMIDKNNKNVNYAFV    120
                     .:   :.******.: :** :: * *.  **  * *:::*.   *  *.**

hsTIA1      EFHEHRHAAAALAAMNGRKIMGKEVKVNWATTPSSQKKDTSSSTVVSTQRSQDHFHVFVG    111
scPUB1      EYHQSHDANIALQTLNGKQIENNIVKINWAFQSQQ-------------SSSDDTFNLFVG    167
            *:*: :.*  ** ::**::* .: **:***   ..             . *:* *::***

hsTIA1      DLSPEITTEDIKAAFAPFGRISDARVVKDMATGKSKGYGFVSFFNKWDAENAIQQMGGQW    171
scPUB1      DLNVNVDDETLRNAFKDFPSYLSGHVMWDMQTGSSRGYGFVSFTSQDDAQNAMDSMQGQD    227
            **. ::  * :: **  *    ..:*: ** **.*:******* .: **:**::.* **

hsTIA1      LGGRQIRTNWATRKPPAPKSTYESNTK---------------------------------    198
scPUB1      LNGRPLRINWAAKRDNNNNNNYQQRRNYGNNNRGGFRQYNSNNNNNMNMGMNMNMNMNMN    287
            *.** :* ***:::    :..*:.. :

hsTIA1      --------------------------------------QLSYDEVVNQSSPSNCTVYCGG    220
scPUB1      NSRGMPPSSMGMPIGAMPLPSQGQPQQSQTIGLPPQVNPQAVDHIIRSAPPRVTTAYIGN    347
                                                    : *.::..: *   *.* *.

hsTIA1      VTSGLTEQLMRQTFSPFGQIMEIRVFPDKGYSFVRFNSHESAAHAIVSVNGTTIEGHVVK    280
scPUB1      IPHFATEADLIPLFQNFGFILDFKHYPEKGCCFIKYDTHEQAAVCIVALANFPFQGRNLR    407
            :    **  :   *. ** *:::: :*:** .*:::::**.** .**:: .  ::*: ::

hsTIA1      CYWGKETLDMINPVQQQNQIGYPQPYGQWGQWYGNAQQIGQYMPNGWQVPAYGMYGQAWN    340
scPUB1      TGWGKERSNFMPQQQQQGG--QPLIMNDQ------------------QQPVMSEQQQQQQ    447
              ****  :::   ***.    *   .:                   * *. .   *  :

hsTIA1      QQGFNQTQSSAPWMGPNYGVQPPQGQNGSMLPNQPSGYRVAGYETQ 386
scPUB1      QQ--QQQQ-------------------------------------- 453
            **  :* *
```

2-15: CLUSTAL O(1.2.4) multiple sequence alignment **[ALS27]**

```
hsSPTLC1      ------------------MATATEQWVLVEMVQA--------------LYEAPAYHLILE   28
scLCB1        MAHIPEVLPKSIPIPAFIVTTSSYLWYYFNLVLTQIPGGQFIVSYIKKSHHDDPYRTTVE   60
                                ::*::  *  .::* :               :.   *:  :*

hsSPTLC1      GILILWIIRLLFSK----TYKLQERSDLTVKEKEELIEEWQPEPLVPPVPKDHPALNYNI   84
scLCB1        IGLILYGIIYYLSKPQQKKSLQAQKPNLSPQEIDALIEDWEPEPLVDPSATDEQSWRV--   118
                ***: *   :**    .    ::  :*: :* : ***:*:***** *  .*. : .

hsSPTLC1      VSGPPSHK-------T------VVNGKECINFASFNFLGLLDNPRVKAAALASLKKYGVG   131
scLCB1        AKTPVTMEMPIQNHITITRNNLQEKYTNVFNLASNNFLQLSATEPVKEVVKTTIKNYGVG   178
              .. * : :       *        : .: :*:** *** *  .  ** .. :::*:****

hsSPTLC1      TCGPRGFYGTFDVHLDLEDRLAKFMKTEEAIIYSYGFATIASAIPAYSKRGDIVFVDRAA   191
scLCB1        ACGPAGFYGNQDVHYTLEYDLAQFFGTQGSVLYGQDFCAAPSVLPAFTKRGDVIVADDQV   238
              :*** ****. ***  **  **:*: *: :::*. .*.:  *.:**::****::..*  .

hsSPTLC1      CFAIQKGLQASRSDIKLFKHNDMADLERLLKEQEIEDQKNPRKARVTRRFIVVEGLYMNT   251
scLCB1        SLPVQNALQLSRSTVYYFNHNDMNSLECLLNELTE-QEKLEKLPAIPRKFIVTEGIFHNS   297
              .: :*:.** *** :  *:**** .** **:*    ::*  :   : *:***.**:: *:

hsSPTLC1      GTICPLPELVKLKYKYKARIFLEESLSFGVLGEHGRGVTEHYGINI-DDIDLISANMENA   310
scLCB1        GDLAPLPELTKLKNKYKFRLFVDETFSIGVLGATGRGLSEHFNMDRATAIDITVGSMATA   357
              * :.*****.*** *** *:*::*::*:****  ***::**:.::    **:  ..* .*

hsSPTLC1      LASIGGFCCGRSFVIDHQRLSGQGYCFSASLPPLLAAAAIEALNIMEENPGIFAVLKEKC   370
scLCB1        LGSTGGFVLGDSVMCLHQRIGSNAYCFSACLPAYTVTSVSKVLKLMDSNNDAVQTLQKLS   417
              *.* ***  * *.:  ***:..:.*****.**   .::. :.*::*:.* . . .*:: .

hsSPTLC1      GQIHKALQGISGLK----VVGESLSPAFHLQLEESTGSREQ-------------------   407
scLCB1        KSLHDSFASDDSLRSYVIVTSSPVSAVLHLQLTPAYRSRKFGYTCEQLFETMSALQKKSQ   477
               .:*.:: . ..*:    *... :* .:****  :  **:

hsSPTLC1      ----------DVRLLQEIVDQCMNR-SIALTQARYLEKEEKCLPPPSIRVVVTVEQTEEE   456
scLCB1        TNKFIEPYEEEEKFLQSIVDHALINYNVLITRNTIVLKQETLPIVPSLKICCNAAMSPEE   537
                        : ::**.***:.: . .: :*:   : *:*.    **:::  ..  : **

hsSPTLC1      LERAASTIKEVAQAVLL----       473
scLCB1        LKNACESVKQSILACCQESNK       558
              *:.*..::*:   *
```

2-16: CLUSTAL O(1.2.4) multiple sequence alignment **[FTD-ALS2]**

```
hsCHCHD10      MP--RGSRSAA--SRPASRPAAP----SAHPPAHPPPSAA---APAPAPSGQPGLMAQMA 49
scMIX17        MARSRGSSRPISRSRPTQTRSASTMAAPVHPQQQQQPNAYSHPPAAGAQTRQPGMFAQMA 60
               *   ***      ***:.  :*      .**  :  *.*      * * : ***::****

hsCHCHD10      TTAAGVAVGSAVGHVMGSALTGAFSGGSSEPSQPAVQQAPTPA-----APQPLQMGPCAY 104
scMIX17        STAAGVAVGSTIGHTLGAGITGMFSGSGSDSAPVEQQQQNMANTSGQTQTDQQLGRTCEI 120
               :*********::**.:*:..:** ***..*: :   **            :      *

hsCHCHD10      EIRQFLDCSTT-QSDLSLCEGFSEALKQCKYYHGLSSLP     142
scMIX17        DARNFTRCLDENNGNFQICDYYLQQLKACQEAARQY---     156
               : *:*  *    :.::.:*: : : ** *:
```

2-17: CLUSTAL O(1.2.4) multiple sequence alignment **[FTD-ALS5]**

```
hsCCNF      -----MGSGGVVHCRCAKCFCYPTKRRIRRRPRNLTILSLPEDVLFHILKWLSVEDILAV     55
scCLN1      MNHSEVKTGLIVT---AKQTYYPIELS-------------NAELLTHY---E------TI     35
                 : :* :*    **   ** :                 ::* *           ::

hsCCNF      RAVHSQLKDLVDNHASVWACASFQELWPSPGNLKLFERAAEKGNFEAAVKLGIAYLYNEG     115
scCLN1      QEYHEEISQ----------NVLVQS-SKTKPDIKLIDQQPEMNPHQ-TREAIVTFLYQLS     83
            :  *.::.:           . .*.   :  ::**:::  * . .: : :  :::**: .

hsCCNF      LSVSDEARAEVNG--LKASRFF--------SLAERLNVGAAPFIWLFIRPPWSVSGSCCK     165
scCLN1      VMTRV-----SNGIFFHAVRFYDRYCSKRVVLKDQAKLVVGTCLWLAA-KTW---GGCNH     134
            : .        **  ::* **:         * :: :: ..  :**     *   *.* :

hsCCNF      AVVHESLRAECQLQRTHKASILHCLGRVLSLFEDEEKQQQAHDLFEEAAHQGC--LTSSY     223
scCLN1      IINNVSIPTGGRFYGPNPRARIPRLSELVH-------YCGGSDLFDESMFIQMERHILDT     187
             : : *: :  ::   :  : :  *..::           . ***:*: .        .

hsCCNF      LLWESDRR----TDVSDPGRCLHSFRKLRDY-----AAKGCWEAQLSLAKACANANQLGL     274
scCLN1      LNWDVYEPMINDYILNVDENCLIQYELYKNQLQNNNSNGKEWSCK--------RKSQSSD     239
            * *:  .       :.   .** .:.  ::      :    *..:        . .* .

hsCCNF      EVRASSE-IVCQLFQASQAVSKQQVFSVQKGLNDTMRYILIDWLVEVATMKDFTSLCLHL     333
scCLN1      DSDATVEEHISSSPQSTGLDGDTTTMDEDEELNSKIKLINL-------------------     280
            :  *: *  :..  *::   ..  .:. :: **..:: * :

hsCCNF      TVECVDRYLRRRLVPRYRLQLLGIACMVICTRFISKE--ILTIREAVWLTD---NTYKYE     388
scCLN1      -----KRFLIDL--SCWQYNLLKFELYEICNGMFSIINKFTNQDQGPFLSMPIGNDINSN     333
                 .*:*       :: :** :    **. ::*    : . :. :*:     *  : :

hsCCNF      DLVRMMGEIVSALEGKIRV-PTVVDYKEVLLTLVPVELRTQHLC-------SFLCELSLL     440
scCLN1      TQTQVFSII---INGIVNSPPSLVEVYKEQYGIVPFILQVKDYNLELQKKLQLASTIDLT     390
              .:::. *   ::* :.  *::*:  :    :**. *:.:.         .: . :.*

hsCCNF      ------------------------HTSLSAYAPARLAAA---ALLLARLTHGQTQPWTTQ     473
scCLN1      RKIAVNSRYFDQNASSSSVSSPSTYSSGTNYTPMRNFSAQSDNSVFSTTNIDHSSPITPH     450
                                    ::* : *:* *  :*     :::  . .::.* * :

hsCCNF      LWDLTGFSYEDLIPCVLSLH---KKCFHDDAP--KDYRQVSLTAVKQRFEDKRYGEISQE     528
scCLN1      MYTFNQFKNESACDSAISVSSLPNQTQNGNMPLSSNYQNMMLE---ERNKENRIPN----     503
            :: :. *. *.   ..:*:    ::  :.: *  .:*::: *    :* :::*  :

hsCCNF      EVLSYSQLCAALGVTQDSPDPPTFLSTGEIHAFLSSPSGRRTKRKRENSLQEDRGSFVTT     588
scCLN1      ------------SSSAEIPQRAKFMTTGIFQ-----------------------------     522
                        . : : *:  .*::** ::

hsCCNF      PTAELSSQEETLLGSFLDWSLDCCSGYEGDQESEGEKEGDVTAPSGILDVTVVYLNPEQH     648
scCLN1      ------------------------------------------------------------     522

hsCCNF      CCQESSDEEACPEDKGPQDPQALALDTQIPATPGPKPLVRTSREPGKDVTTSGYSSVSTA     708
scCLN1      ------------------------------------------------------------     522

hsCCNF      SPTSSVDGGLGALPQPTSVLSLDSDSHTQPCHHQARKSCLQCRPPSPPESSVPQQQVKRI     768
scCLN1      --------NTGELTNRASSISLSLRNHNSSQL----------------------------     546
                    . * * : :* :**.  .*..

hsCCNF      NLCIHSEEEDMNLGLVRL     786
scCLN1      ------------------     546
```

2-17 (continued): CLUSTAL O(1.2.4) multiple sequence alignment **[FTD-ALS5]**

```
hsCCNF      MGSGGVVHCRCAKCFCYPTKRRIRRRPRNLTIL-SLPEDVLFHILKWLSVEDILAVRAVH    59
scCLN2      -------------------MASAEPRPRMGLVINAKPDYYPIE-----------------    24
                                   . ***   :: : *:   :.                 

hsCCNF      SQLKDLVDNHASVWACASFQELWPSPGNLKLFERAAEKGNFEAAVKLGIAYLYNEGLSVS    119
scCLN2      ------------------------------------------------------------    24

hsCCNF      DEARAEVNGLKASRFFSLAERLNVGAAPFIWLFIRPPWSVSGSCCKAVVHESLRAECQLQ    179
scCLN2      ------------------------------------------------------------    24

hsCCNF      RTHKASILHCLGRVLSLFEDEEKQQQAHDLFEEAAHQGCLTSSYLLWESDRRTDVSDPGR    239
scCLN2      -------------------------------------------------------LSNAE    29
                                                                    . ..

hsCCNF      CLHSFRKLRDYAAKGCWEAQLSLAKACANANQLGLEVRASSEIVCQLFQASQAVSKQQVF    299
scCLN2      LLSHFEMLQEYHQEIST---NVIAQ------------------SCKFKPNPKLIDQ----    64
             *  *. *::*  : .      :*:                   *::    : :.:    

hsCCNF      SVQKGLN-DTMRYILIDWLVEVATMKDFTSLCLHLTVECVDRYLRRRLVPRYRLQLLGIA    358
scCLN2      --QPEMNPVETRSNIITFLFELSVVTRVTNGIFFHSVRLYDRYCSKRIVLRDQAKLVVAT    122
              *  :*    *  :* :*.*::.:. .*.  :. :*.  ***  :*:* * : :*:  :

hsCCNF      CMVICTRFISKEILTIREAVWLTDNTYKYEDLVRMMGEIVSALEGKIRVPTVVDYKEVLL    418
scCLN2      C------------------LWLAAKTWGGCN--HIINNVVIPTGGRFYGPNPRAR-----    157
            *                  :**: :*:   :  :::.::*    *::  *.

hsCCNF      TLVPVELRTQHLCSFLCELSLLHTSLSAYAPARLAAAALLLARLTHGQTQPWTTQLWDLT    478
scCLN2      --IPRLSELVHYCGD---GQVFDE-------------SMFLQ-M---ERHILDTLNWNIY    195
              :*   .  * *.     .::.              :::*  :   : :   *  *::

hsCCNF      GFSYEDLIPCVLSLHKKCFHDDAP-----------KDYRQVSLTAV-KQRFEDKRYGEIS    526
scCLN2      EPMIND---YVLNVDENCLMQYELYENQVTYDKQCSEKRQSQLSQDSDATVDERPYQNEE    252
                :*    **.:.::*: :              .: ** .*:   .  .::: * : .

hsCCNF      -------------------------QEEVLSYSQLCAALGVTQDSPDPPTFLSTGEIHAF    561
scCLN2      EEEEDLKLKIKLINLKKFLIDVSAWQYDLLRYELFEVSHGIF----------------S-    295
                                     * ::* *. : .: *:                 :

hsCCNF      LSSPSGRRTKRKRENSLQEDRGSFVTTPTAELSSQEETLLGSFLDWSLDCCSGYEGDQES    621
scCLN2      -----------IINQFTNQDHGPFLMTPMTSESKNGEIL-STLM----------------    327
                         ::  ::*:* *: ** :. *.: * * .:::

hsCCNF      EGEKEGDVTAPSGILDVT---------------------------------VVYLNPEQH    648
scCLN2      ----NGIVSIPNSLMEVYKTVNGVLPFINQVKEYHLDLQRKLQIASNLNISRKLTISTPS    383
                :* *: *..:::*

hsCCNF      CCQESSDEEACPEDK----------------------------GPQDPQALALDTQIP--    678
scCLN2      CSFENSNSTSIPSPASSSQSHTPMRNMSSLSDNSVFSRNMEQSSPITPSMYQFGQQQSNS    443
            *. *.*:. : *.                              .*  *.   :. *

hsCCNF      ------------ATPGPKPLVRTSREPGKDVTTSGYSSV------------------STA    708
scCLN2      ICGSTVSVNSLVNTNNKQRIYEQITGPNSNNATNDYIDLLNLNESNKENQNPATAHYLNG    503
                         * . : : .    *..: :*..* .:                   ..

hsCCNF      SPTSSVDGGLGALPQPTSVLSLDSDSHTQPCHHQARKSCLQCRPPSPPESSVPQQQVKRI    768
scCLN2      GPPKTSFINHGMFPSPTGTINSGKSSSASSL----------------------------I    535
            .* .:   . * :*.**..:. ...* :.                              *
```

```
hsCCNF      NLCIHSEEEDMNLGLVRL-   786
scCLN2      ---------SFGMGNTQVI   545
                     .:.:* .::
```

2-17 (continued): CLUSTAL O(1.2.4) multiple sequence alignment **[FTD-ALS5]**

```
hsCCNF          MGSGGVVHCRCAKCFCYPTKRRIRRRPRNLTILSLPEDVLFHILKWLSVEDILAVRAVHS     60
scCLN3          ------------------------------------------------------------     0


hsCCNF          QLKDLVDNHASVWACASFQELWPSPGNLKLFERAAEKGNFEAAVKLGIAYLYNEGLSVSD     120
scCLN3          ------------------------------------------------------------     0


hsCCNF          EARAEVNGLKASRFFSLAERLNVGAAPFIWLFIRPPWSVSGSCCKAVVHESLRAECQLQR     180
scCLN3          ------------------------------------------------------------     0


hsCCNF          THKASILHCLGRVLSLFEDEEKQQQAHDLFEEAAHQGCLTSSYLLWESDRRTDVSDPGRC     240
scCLN3          -------------MAILK----------------------DTIIRYANARYATASGTST-     24
                             :::::                      .: : : . * : .*. .

hsCCNF          LHSFRKLRDYAAKGCWEAQLSLAKAC-------ANANQLGLEVRASS-----------EI     282
scCLN3          ----ATAASVSAASCPNLPLLLQKRRAIASAKSKNPNLVKRELQAHHSAISEYNNDQLDH     80
                     .  . :* .* :  * * *          * * :  *::*             :

hsCCNF          VCQLFQASQAVSKQQVFSVQKGLNDTMRYILIDWLVEVATMKDFTSLCLHLTVECVDRYL     342
scCLN3          YFRLSHTERPLYNLTNFNSQPQVNPKMRFLIFDFIMYCHTRLNLSTSTLFLTFTILDKYS     140
                  :* ::.: : :   *. *  :* .**::::*:::   *  ::::  *.**.  :*:*

hsCCNF          RRRLVPRYRLQLLGIACMVICTRFISKEIL--TIREAVWLTDNTYKYEDLVRMMGEIVSA     400
scCLN3          SRFIIKSYNYQLLSLTALWISSKFWDSKNRMATLKVLQNLCCNQYSIKQFTTMEMHLFKS     200
                 * ::  *. ***.::.: *.::* ..:    *::    *  * *. :::. *  .:..:

hsCCNF          LEGKIRVP-TVVDYKEVLLTL------------VPVELRTQ-------------------     428
scCLN3          LDWSICQSATFDSYIDIFLFQSTSPLSPGVVLSAPLEAFIQQKLALLNNAAGTAINKSSS     260
                *: .*    *. .* :::*              .*:*   *

hsCCNF          ------------HLCSFLCELSLLHTSLSA-YAPARLAAAALLLARLTHGQTQPWTTQLW     475
scCLN3          SQGPSLNINEIKLGAIMLCELASFNLELSFKYDRSLIALGAINLI---KLSLNYYNSNLW     317
                              . :****: :: .**  *  : :* .*: *    : . : :.::**

hsCCNF          DLTGFSYEDLIPCVLSLHKKCFHDDAPKDYRQVSLTAVKQRFEDKRYGEISQEEVLSYSQ     535
scCLN3          ENINLALEE----------NCQDLDIK-------------------LSEISNT-------     341
                :  .:: *:          :* . *                      .***:

hsCCNF          LCAALGVTQDSPD-PPTFLSTGEIHAFLSSPSGRRTKR----KRENSLQEDRGSFVTTPT     590
scCLN3          ---LLDIAMDQNSFPSSFKS-----KYLNSNKTSLAKSLLDALQNYCIQLKLEEFYRS--     391
                    *.:: *. . * :* *      :*.* .   :*      :: .:* .  .*  :

hsCCNF          AELSSQEETL-LGSFLDWSLDCCSGYEGDQESEGEKEGDVTAPSGILDVTVVYLNPEQHC     649
scCLN3          QELETMYNTIFAQSFDSDSLTCVYSNATTPKS-----A------TVSSAATDYFSDHTHL     440
                 **.:  :*:   ** . ** *  .     :*     .       : ..:. *:. . *

hsCCNF          CQESSDEEACPEDKGPQDPQALALDTQIPATPGPKPLVRTSRE------PGK---DVTTS     700
scCLN3          RRLTKDSISPPFAFTP---------T--SSSSSPSPFNSPYKTSSSMTTPDSASHHSHSG     489
                 : :.*. : *    *         *   :: .*.*:    :       *..   .  :.

hsCCNF          GYSSVSTASPTSSVDGGLGALPQPTSVLSLDSDSHTQPCHHQAR----------KSCLQC     750
scCLN3          SFSST-----QNSFKRSL-SIPQNSSIFWPSPLTPTTPSLMSNRKLLQNLSVRSKRLFPV     543
                .:**.      .*.. .* ::** :*::  .  : * *.  . *          *  :

hsCCNF          RPPSPPE-SSVPQQQVKRINLCIHSE-EEDMNLGLVRL 786
scCLN3          RPMATAHPCSAPTQLKKRSTSSVDCDFNDSSNLKKTR- 580
                ** :  . .*.* *  ** . .:..: ::. **  .*
```
